# Sex Differences and the Role of Estradiol in Mesolimbic Reward Circuits and Vulnerability to Cocaine and Opiate Addiction

**DOI:** 10.3389/fnbeh.2020.00074

**Published:** 2020-05-20

**Authors:** Saurabh S. Kokane, Linda I. Perrotti

**Affiliations:** Department of Psychology, University of Texas at Arlington, Arlington, TX, United States

**Keywords:** estrogen, dopamine, female, withdrawal, mesolimbic reward system

## Abstract

Although both men and women become addicted to drugs of abuse, women transition to addiction faster, experience greater difficulties remaining abstinent, and relapse more often than men. In both humans and rodents, hormonal cycles are associated with females’ faster progression to addiction. Higher concentrations and fluctuating levels of ovarian hormones in females modulate the mesolimbic reward system and influence reward-directed behavior. For example, in female rodents, estradiol (E2) influences dopamine activity within the mesolimbic reward system such that drug-directed behaviors that are normally rewarding and reinforcing become enhanced when circulating levels of E2 are high. Therefore, neuroendocrine interactions, in part, explain sex differences in behaviors motivated by drug reward. Here, we review sex differences in the physiology and function of the mesolimbic reward system in order to explore the notion that sex differences in response to drugs of abuse, specifically cocaine and opiates, are the result of molecular neuroadaptations that differentially develop depending upon the hormonal state of the animal. We also reconsider the notion that ovarian hormones, specifically estrogen/estradiol, sensitize target neurons thereby increasing responsivity when under the influence of either cocaine or opiates or in response to exposure to drug-associated cues. These adaptations may ultimately serve to guide the motivational behaviors that underlie the factors that cause women to be more vulnerable to cocaine and opiate addiction than men.

## Introduction

Drug addiction or substance use disorder is a chronic, relapsing, neuropsychiatric illness characterized by a loss of control over drug seeking and intake, persistent drug craving, and high motivation to take the drug (Reid et al., 2012). Here we provide an updated review, of the literature in which we discuss sex differences in the development of cocaine and opiate addiction, with an emphasis on the influence of estradiol (E2) on signaling within the mesolimbic reward circuit. We provide an overview of the mechanisms of the mesolimbic reward circuit and have described in detail how E2 influences it at the circuit level and also at the level of dopaminergic signaling. Furthermore, we have included discussions of molecular mechanisms underlying the sex differences in the behavioral responses to cocaine and opiates and have demonstrated the role of E2 in the modulation of these mechanisms. Taken together, this review demonstrates the link between sex differences in cocaine and opiate addiction and the role of E2 as a major driver of these sex differences. Many excellent reviews have been published on sex differences in addiction and drug abuse. Some of the most recent of these have detailed sex differences in the balance between dorsal and ventral striatal circuits (Becker, 2016); quantitative, population, and mechanistic sex differences in addiction (Becker and Koob, 2016); basic biological differences between males and females that influence addictive behaviors (Becker et al., 2017); sex differences in biology, epidemiology, and treatment of substance use disorder (McHugh et al., 2018), and, most recently, a comprehensive review which highlights sex differences in the neural mechanisms and developmental events influencing addiction vulnerability (Becker and Chartoff, 2019).

### Sex Differences in Addiction to Cocaine

Cocaine use disorder is a serious public health concern. In the United States, approximately 2.2 million people report regular use of cocaine and 1 million individuals met criteria for cocaine use disorder in the past year (Substance Abuse and Mental Health Services Administration, 2018). Although, overall, more men use and are addicted to cocaine than women, women exhibit a more rapid progression from initial cocaine use to dependence than men (Kosten et al., 1985; Brady and Randall, 1999). Women also report experiencing enhanced positive subjective effects (feelings of euphoria) of cocaine than men. The enhancement of positive subjective effects is speculated to be the reason women more rapidly progress through the stages of addiction than men (reviewed in Becker and Hu, 2008). During periods of abstinence, women report experiencing higher levels of cocaine craving and relapse rates than men. Lastly, during bouts of relapse women take larger amounts of cocaine than men (Kosten et al., 1985; Brady and Randall, 1999; Robbins et al., 1999; Chen and Kandel, 2002; Gallop et al., 2007; Ignjatova and Raleva, 2009). Collectively, these data indicate that women may be more severely affected by cocaine use than men.

### Sex Differences in Addiction to Opiates

Opioid use disorder (OUD) has reached epidemic proportions, claiming ∼115 lives daily to opioid overdoses in the US alone (Substance Abuse and Mental Health Services Administration, 2015; The, 2017; Volkow et al., 2019). Opioids acutely attenuate perceptions of pain and induce euphoria and relaxation in users. Despite the acute benefits, long-term opioid use can lead to the development of OUD (The, 2017; Volkow et al., 2019). Individual differences in addiction liability exist, and behavioral traits like impulsivity and risk-taking behavior often heighten the risk for users to transition from recreational drug use to drug dependence (Kreek et al., 2005; Belin et al., 2008). The influence of sex/gender in the physiological/psychological risks for developing OUD has also been reported (Fillingim et al., 2009; Green et al., 2009; Back et al., 2010). Overall, more men than women report lifetime and past-year use of all opioid drugs. However, women are more likely to report the non-medical use of prescription opioids as their primary drug of abuse (Center for Behavioral Health Statistics and Quality, 2015). This is likely because women are more likely to suffer from chronic pain conditions (Darnall et al., 2012), be prescribed prescription pain relievers, and use them for longer time periods than men (Berkley, 1997; Gureje et al., 1998; Fillingim et al., 2009; Mogil, 2012; Centers for Disease Control and Prevention, 2013; Substance Abuse and Mental Health Services Administration, 2014; Serdarevic et al., 2017). In addition, compared to men, women (*a*) are more likely to use opioids for non-medical conditions (Green et al., 2009; Hemsing et al., 2016; Marsh et al., 2018); (*b*) transition more rapidly from casual to non-medical use of prescription opioids to opioid dependence; (*c*) experience higher levels of craving and relapse during abstinence; (*d*) consume larger amounts of drug during relapse; and (*e*) are less likely to seek treatment for their opioid addiction (Kosten et al., 1985; Brady and Randall, 1999; Ignjatova and Raleva, 2009; Bobzean et al., 2014a). In addition, during periods of abstinence, women report higher numbers of distressing physical/affective withdrawal symptoms compared to men (Soldin et al., 2011; Fox et al., 2013; Fernandez-Montalvo et al., 2017; Dunn et al., 2018). While, men are more likely to die from opioid overdose than women, overdoses related to opioids have greatly increased in women compared to men: from 1999 to 2017 opioid overdose-related deaths increased in women by 471% as compared to 218% among men (Paulozzi et al., 2011; Serdarevic et al., 2017; VanHouten et al., 2019). Therefore, it is not surprising that several studies identify women as one of the most vulnerable subpopulations for non-medical prescription opioid use and abuse (Fillingim et al., 2009; Green et al., 2009; Back et al., 2010).

## The Role of the Mesolimbic Reward System in Addiction

**FIGURE 1:**
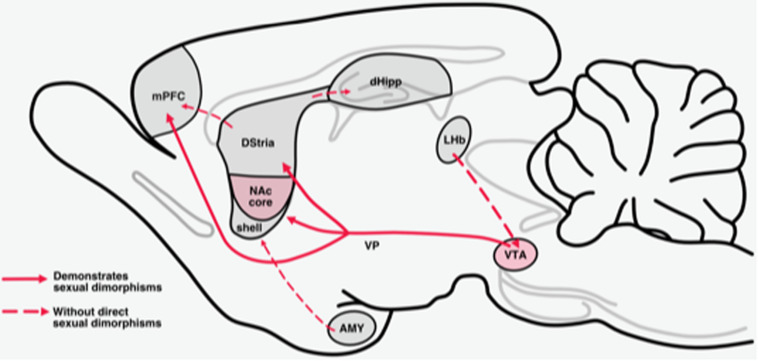
Mesolimbic reward circuits. The mesolimbic pathway originates with dopaminergic cell bodies in the VTA. Dopamine cell bodies in the VTA are tonically inhibited by GABAergic neurons in the VTA. This pathway projects primarily to the NAc, dorsal striatum (Dstria), and medial prefrontal cortex (mPFC). Sexual dimorphisms that are established for projections within these pathways and are delineated with a solid line.

The mesolimbic reward system is necessary for organisms to engage in reinforcing behaviors and to motivate actions that produce rewarding feelings of pleasure (Gardner, 2011). Midbrain dopamine (DA) neurons that arise in the ventral tegmental area (VTA) and substantia nigra project to forebrain regions (striatum, prefrontal cortex, hippocampus, and amygdala) that regulate the motivational and cognitive processes necessary for organisms to engage in behaviors that produce rewarding feelings of pleasure (Wise, 2004) (Figure 1). These pathways modulate information flow through the limbic system to regulate and/or promote behaviors related to survival and perpetuation of the species (i.e., feeding, drinking, mating, maternal and paternal behaviors, and social interaction) (White and Milner, 1992; Gardner, 2011). Chronic drug use produces a persistent enhanced activation of these systems which results in long-term structural and functional changes (Nestler, 2016). Drug-induced enhanced activation of these systems, in turn, alters the motivational and cognitive processes arising from these systems in such a way that the drive to obtain and use drugs supersedes other drives (Anselme, 2009; Goldstein and Volkow, 2011; Buchta and Riegel, 2015; Zorrilla and Koob, 2019).

### VTA in Drug Addiction

The VTA contains two major classes of DA projection systems: the mesolimbic system which regulates rewarding motivational processes including feeding, drinking, sex, drug consumption and continued drug use, and the mesocortical DA system which regulates cognitive/executive functions such as working memory, drug-cue associations, cue-induced reinstatement of drug use (Figure 1) (Le Moal and Simon, 1991; Bjorklund and Dunnett, 2007).

### Striatal Complex in Drug Addiction

The striatal complex, generally speaking, gathers inputs from the neocortex, and then sends projections to other nuclei of the basal ganglia which ultimately reach cortical areas implicated in motor planning and execution. The striatum is a structure that is highly conserved across species and critically important for broad range of cognitive, sensorimotor, and limbic-related functions. The striatal complex is anatomically divided into the ventral striatum (nucleus accumbens) and dorsal striatum (caudate putamen) (Voorn et al., 2004; Yin et al., 2004).

#### Dorsal Striatum

The dorsal striatum (DS) can be subdivided into dorsomedial striatum (DMS), which is vital for goal-directed learning, and the dorsolateral striatum (DLS) implicated in stimulus-response learning (Yin et al., 2005, 2006). Compulsive responding to drug-associated cues is established when the DS is engaged by the intrastriatal loops between the NAc and DS. Thereafter, exposure to drug-associated cues induces activation of the DS without the presence of the drug. Such activation of DS neurons may induce craving for the drug and has been proposed to be the initiator of drug seeking and relapse (Koob and Volkow, 2016).

#### Nucleus Accumbens (a.k.a. Ventral Striatum) in Addiction

The NAc is a heterogeneous structure and is subdivided into shell and core components which are chemoarchitecturally and functionally distinct (Zahm and Brog, 1992; Groenewegen et al., 1999; Di Chiara, 2002). The central area of the NAc, called the NAc core, is distinct and is surrounded medially, ventrally, and laterally by the NAc shell. The differences between the NAc core and NAc shell are defined by a number of histochemical, electrophysiological, connectional, and cellular criteria (Zahm and Brog, 1992; Groenewegen et al., 1999; Di Chiara, 2002). Anatomically, the NAc core/shell subdivisions can be differentiated in terms of the input they receive from prefrontal cortical regions: The NAc core receives the majority of its prefrontal input from the prelimbic region of the cortex and lateral orbitofrontal cortex; the NAc shell receives its cortical input from infralimbic cortex and medial lateral orbitofrontal cortex (Berendse et al., 1992; Wright and Groenewegen, 1996). These accumbal subregions are also dissociable in terms of their pallidal/nigral projection outputs: The core projects predominantly to the substantia nigra while the shell targets the pallidum and the VTA (Heimer et al., 1991; Zahm and Brog, 1992; Zahm and Heimer, 1993; Ikemoto, 2007). There is some evidence to suggest communication between MSNs within the ventral striatum. Within the NAc, the core and shell share important intra-structural connections which may be important for the combination of diverse limbic inputs to be later integrated for output to other structures (van Dongen et al., 2005, 2008).

The NAc core is critically involved in the development and expression of addiction-related behaviors (Koob and Volkow, 2010) and is recruited in Pavlovian conditioning (Koos and Tepper, 1999; Lof et al., 2007; Sunsay and Rebec, 2014). For example, typically, NAc core interacts with brain regions associated with motor circuitry, thus coordinating behavioral output, while the shell interacts with limbic and autonomic brain regions, indicating significant regulation of reward, emotional, and visceral responses to stimuli (Heimer and Alheid, 1991; Zahm and Brog, 1992; Everitt et al., 1999). As such, the shell is suspected to mediate the reinforcing properties of novelty, feeding behavior, rewarding substances and stimuli which induce drug relapse, while the core seems to play a role in spatial learning, conditioned responses, responses to motivational stimuli, and impulsive choices. Together, the NAc core and shell control the enactment and reinforcement of conditioned behaviors through interaction with reward circuitry (Meredith et al., 2008). In this way, it is generally accepted that the NAc shell is more involved in shorter-term aspects of addiction, for instance reward; whereas, the NAc core plays a role in longer lasting reward-directed behaviors (Ito et al., 2004; Meredith et al., 2008).

### Prefrontal Cortex in Drug Addiction

The VTA sends dopaminergic (DA) projections to the medial prefrontal cortex (mPFC). These DA projections activate the glutamatergic systems of the prefrontal cortex. Glutamatergic projections from the prefrontal cortex (PFC) directly activate mesocortical DA neurons in the VTA and exert excitatory control over DA cell firing and release in the PFC (Geisler and Wise, 2008). The PFC also sends glutamatergic projections to the dorsal and ventral striatum which modulates the control of pallidal/nigral pathways. In this way the PFC is in a good position to regulate salience and conditioned behavior in response to salient stimuli (Koob and Volkow, 2016). Protracted abstinence from drugs of abuse leads to over activation of the glutamatergic systems that induce strong craving-like responses via glutamatergic activation of the NAc (McFarland and Kalivas, 2001; De Witte et al., 2005), thus indicative of this circuit’s importance in cravings associated with drugs of abuse. Drug-induced reinstatement also involves glutamatergic projections to the NAc that modulate DA release within the NAc (Koob and Volkow, 2016).

### Hippocampus in Addiction

The hippocampus has been implicated in the formation of drug-context memories, drug-cue associations, and reconsolidation of drug memories. In addition, the hippocampus has been implicated in reinstatement of drug-taking behavior leading to relapse via cue and contextual triggers (Kutlu and Gould, 2016).

### Amygdala in Addiction

The basolateral amygdala (BLA) plays a critical role in the response to natural reward and drug-associated cues. Anatomically, the BLA receives DA inputs from the VTA and provides outputs to neurons in the NAc (Floresco et al., 1998, 2001; Bissière et al., 2003; Ford et al., 2006). Because the BLA serves as an interface between VTA DAergic inputs and outputs to the PFC and NAc, it is well positioned to subserve associative memory functions. Through the convergence of DAergic inputs with sensory-associative information, BLA neurons encode emotionally salient memories (Grace and Rosenkranz, 2002; Rosenkranz and Grace, 2002). The BLA is implicated in the associative properties of opiate-related learning (Fuchs and See, 2002). Increases in activity in adjacent central nucleus of the amygdala (CeA) are associated with the anxiety-like effects of acute withdrawal and the increased drug intake associated with dependence (Koob and Le Moal, 2008).

## Sex Differences on Mesolimbic Reward System Function

In this section, we discuss sex differences that have been identified and described within the mesolimbic reward system. Sexual dimorphisms have been established in both the underlying organization of the midbrain DA circuitry, as well as the influence of estradiol on DA activity (Figure 1).

### Sex Differences in the VTA

Sex and levels of ovarian hormones influence DA cells in the VTA (Morissette et al., 2008; Gillies and McArthur, 2010; Johnson et al., 2010). Female rodents have a significantly greater proportion of DA neurons in the VTA compared to their male counterparts (Kritzer and Creutz, 2008). In addition, sex differences in the shape and volume of the VTA as well as in the distribution and size of DA cell populations have been identified (McArthur et al., 2007).

Although baseline firing activity of DA cells in the VTA of male and female rodents is reported to be equivalent (Locklear et al., 2017; Rincon-Cortes and Grace, 2017), the activity of VTA-DA neurons appears to be sensitive to circulating levels of estradiol (E2). For example, basal firing rates of DA neurons of the VTA vary during the different phases of the rodent estrous cycle: DA firing rates are highest in estrus, lowest in proestrus, and intermediate in diestrus (Zhang et al., 2008). Moreover, E2 replacement to ovariectomized (OVX) rats influences firing rate, spontaneous activity, DA release, DA transporter activity, and overall responsiveness of striatal neurons to DA (Zhang et al., 2008; Calipari et al., 2017). In addition, DA receptor auto-inhibition is also E2 sensitive, demonstrating greater inhibition with increasing levels of E2 over the estrous cycle and increased inhibition in OVX-E2 treated mice (Vandegrift et al., 2017). Overall, the ability of E2 to influence activity of VTA-DA neurons strongly suggests the involvement of locally expressed estrogen receptors (Shughrue et al., 1997; Creutz and Kritzer, 2002; Milner et al., 2010).

### Sex Differences in the Striatal Complex

The GABAergic medium spiny neuron (MSN) is the predominant striatal neuron type (∼95%) (Gerfen and Surmeier, 2011). GABAergic MSNs of the DS are capable of influencing both motor and cognitive behaviors via their projections to other brain regions (Yager et al., 2015). GABAergic MSNs work intricately to integrate inputs from a number of different brain areas to determine the final output of the striatum. For example, GABAergic MSNs of the NAc make inhibitory connections with cells in the ventral pallidum and VTA, and receive excitatory input from the prefrontal cortex, ventral subiculum of the hippocampus and basolateral nucleus of the amygdala (Sesack et al., 2003; Kauer and Malenka, 2007). MSNs also receive other inputs (predominately excitatory) from multiple brain regions implicated in mediating striatal function (Russo and Nestler, 2013; Scofield et al., 2016).

Robust sex differences and hormone sensitivity in the NAc core are well documented (Becker, 1999; Becker and Hu, 2008; Yoest et al., 2014). Reports of sex differences/hormone sensitivity in NAc shell are less robust and/or more variable compared to core and likely depend upon interactions with other environmental influences (Forlano and Woolley, 2010; Brancato et al., 2017). The sex differences in the NAc core appear to be mediated primarily via influences on excitatory synaptic and electrophysiological properties of NAc neurons and striatal terminals (Mermelstein et al., 1996; Wissman et al., 2011; Dorris et al., 2015). In addition, sexual dimorphisms in synaptic properties and dendritic spine density of GABAergic MSNs in the NAc core have also been identified. In females, GABAergic MSNs of NAc core have anatomically larger spines and higher dendritic spine density than in males (Forlano and Woolley, 2010) and the frequency of mEPSCs in the core is higher in females than males (Wissman et al., 2011). In both the core and shell there is no evidence for sex differences in the number of DA neurons (Forlano and Woolley, 2010; Wissman et al., 2012). Other neuroanatomical attributes such as MSN soma size, cellular density and gross region volume have not been found to be sexually dimorphic (Meitzen et al., 2011; Wong et al., 2016). Electrophysiological properties of GABAergic MSNs in the core change across the estrous cycle (Proano et al., 2018). For example, during diestrus, the excitatory synaptic input onto these MSNs decreases in magnitude, while intrinsic excitability increases. In other words, mESPC frequency and amplitude are decreased during diestrus compared to other estrous cycle phases, while properties such as action potential rheobase, threshold, input resistance, and resting membrane potential change to increase cellular excitability (Proano et al., 2018). During proestrus and estrus excitatory synaptic input increases and intrinsic excitability decreases. Frequency and amplitude of mEPSCs are also increased compared to diestrus phase (Proano et al., 2018). These findings demonstrate the likelihood that higher and lower levels of E2 differentially regulate the electrophysiological properties of GABAergic MSNs of the NAc core. Based on these data, it can be inferred that lower levels of E2 during diestrus may permit tonic activation of GABAergic MSNs while higher levels of E2 during proestrus and estrus induces GABA release.

The major target of the VTA-DA projections is striatal GABAergic MSNs (Figure 1). Within the NAc, subpopulations of MSNs are distinguished based on expression of DA receptor subtype and connectivity to other structures (Gerfen et al., 1990). These include, D1 receptors which have an excitatory influence on movement and reward project directly to the substantia nigra reticulata and D2 receptors which most often have inhibitory effects and project to the external segment of the globus pallidus (Deng et al., 2006; Garcia-Carmona et al., 2015; Volkow and Morales, 2015). These neurons can be further defined by their transcriptional profiles: D1substance P and dynorphin and D2 enkephlin (Gerfen et al., 1990; Lobo and Kennedy, 2006; Heiman et al., 2008). It has been suggested that 5–15% of dorsal striatum MSNs can express both D1 and D2 receptors (Lester et al., 1993; Bertran-Gonzalez et al., 2008; Perreault et al., 2011). D1-MSNs are equally distributed throughout the core and shell (Gangarossa et al., 2013). D2-MSNS are homogeneously distributed in the core but in the shell, they are more expressed in the medial and ventral shell (Gangarossa et al., 2013).

The striatum of male rats contains about 10% more D1 DA receptors than that of intact female or OVX rats. Generally speaking, there are no sex differences in the number or binding characteristics of striatal D2 DA receptors (Hruska and Silbergeld, 1980; Levesque and Di Paolo, 1988), however, one experiment reported female rats had fewer D2 receptors than males (Miller, 1983). Interestingly, E2 rapidly downregulates D2 DA receptor binding in the striatum of females (Bazzett and Becker, 1994).

Sex differences and E2 sensitivity of striatal DA kinetics have been well documented (Yoest et al., 2018). For example, administration of E2 to OVX rats increases DA release, turnover, and DA uptake (Becker and Ramirez, 1981a; Becker and Beer, 1986; Di Paolo, 1994). In addition, E2 acutely increases DA receptor density and DA binding (Di Paolo et al., 1985; Levesque and Di Paolo, 1989; Di Paolo, 1994; Shieh and Yang, 2008). Evidence from studies using intact rodents add to the above studies and clearly demonstrate sex differences in baseline DA activity and stimulated DA activity in the striatum (Becker, 1999; Becker and Hu, 2008; Becker et al., 2012). More specifically, female rats exhibit greater basal concentrations of DA and stimulated DA concentrations in the striatum compared to those of males (Castner et al., 1993; Walker et al., 2000). The ratio of levels of striatal DOPAC/DA (a measure of neurotransmitter turnover) are highest during the proestrus stage of the estrous cycle as compared to the other stages of the cycle, suggesting a greater magnitude of DA turnover when circulating levels of E2 are high (Xiao and Becker, 1994).

## Sex Differences in the Behavioral Response to Drugs of Abuse

Like humans, female rodents escalate administration of cocaine and opioids more rapidly than males (Becker and Chartoff, 2019). Females rodents also demonstrate higher motivation to consume cocaine and opioids than their male counterparts (Becker and Chartoff, 2019). In addition, females consume larger quantities of drug (under some but not all conditions) and experience increased rewarding effects of cocaine and morphine compared to their male counterparts (reviewed in Becker and Koob, 2016). Post abstinence, female rodents consume greater amounts of the drug and demonstrate greater dysregulation of drug intake when given free access to drugs than males (Lynch and Taylor, 2004, 2005; Fuchs et al., 2005; Kosten and Zhang, 2008). Females also demonstrate increased responsivity toward drug taking under stress than males indicating a higher intensity of negative physical and psychological withdrawal symptoms and therefore, higher relapse probability (Fox and Sinha, 2009). Lastly, female rodents more readily reinstate drug use after a period of abstinence in the absence of any reinforcing cues when compared to males (Anker and Carroll, 2010; Buffalari et al., 2012). Taken all together, these data demonstrate sex differences in preclinical rodent models of drug administration/reward and recapitulate and extend findings from the clinical literature. Specifically, female rodents are decisively more vulnerable to developing addiction-like behaviors after exposure to drugs of abuse than males. Although animal models of drug addiction do not emulate exactly what happens in humans, the assessment of the behaviors that occur after ingestion of drugs of abuse have demonstrated good face validity in that they allow for the objective understanding of specific signs and symptoms of the addiction process. They also allow quantification of psychological constructs like drug reward and affective states. Ultimately, much of the extant scientific literature involved in the understanding of underlying neurobiology and pathophysiology of drug addiction comes from the use of animal models of drug addiction. With regard to this review, almost all evidence-based inferences to demonstrate the interaction between estradiol and mesolimbic reward circuitry toward the vulnerability of women to drug addiction have been derived from the preclinical rodent literature. This further elaborates the validity of the use of these rodent models toward understanding the basis of sex differences in drug addiction.

### The Menstrual and Estrous Cycle Influence Addictive Behaviors

**FIGURE 2:**
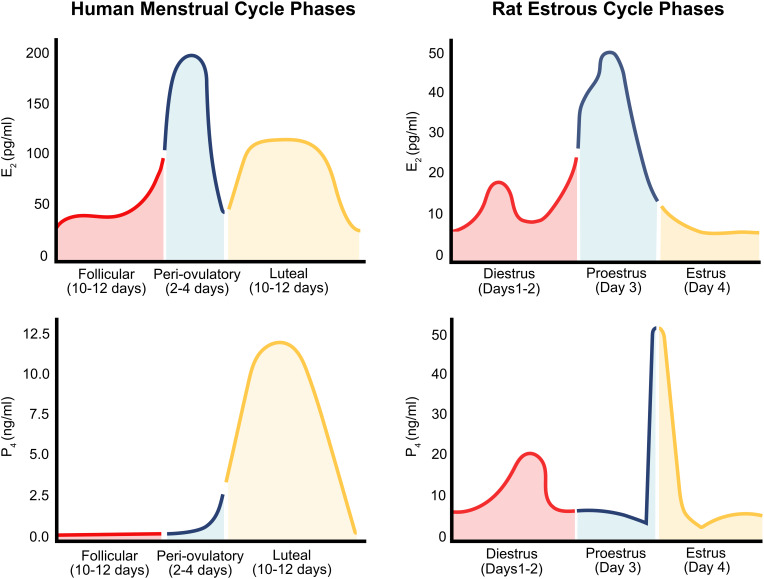
The human menstrual cycle and rat estrous cycle. The human menstrual cycle **(left)** occurs over 28 days and is comprised of fluctuating levels of E2 (E2); (top) and progesterone (P4; bottom). Levels of E2 rise to a peak during the peri-ovulatory phase. E2 levels then drop, rise, and plateau between during the mid-luteal phase (approximately Days 14–28). Progesterone (P4) levels begin to at the end of the ovulation (Days 0–14) and reach their peak during the mid-luteal phase of the cycle (approximately Day 7). The rat estrous cycle **(right)** is similar to the human menstrual cycle but occurs over a 4/5-day period. Four phases; metestrus, diestrus, proestrus, and estrus comprise the rat estrous cycle. Estrogen levels (top) peak toward the end of diestrus and beginning of proestrus. Progesterone levels (bottom) reach a peak during mid-proestrus.

In females, the phase of the menstrual/estrous cycle and the release of reproductive hormones associated with each phase, influences synaptic transmission, sex-specific motivated behaviors, as well as motivation related to drug-taking and addiction-related behaviors (Figure 2). Therefore, it is essential that the hormonal conditions of women be taken into consideration when discussing sex differences in addiction.

The human menstrual cycle has a 28-day duration and consists of follicular, periovulatory, and luteal phases. During the follicular phase (10–12 days), estradiol (E2) is secreted from the ovary as the follicle develops and circulating concentrations of E2 increase daily. The next phase is the peri-ovulatory phase (2–4 days) during which a surge of E2 prompts the release of luteinizing hormone from the pituitary that induces ovulation. The next phase is the luteal phase (10–12 days) which is characterized by the release of relatively high concentrations of both E2 and progesterone from the remains of the ruptured follicle (corpus luteum). Menstruation occurs at the end of the luteal phase after the fall in progesterone and E2 secretion once the corpus luteum has regressed (unless pregnancy occurs). Hormone levels are at their lowest points during menstruation indicating the beginning of the next follicular phase (Becker et al., 2005). Rats and mice have a 4–5 days estrous cycle comprised of phases that function similarly to the phases in the human menstrual cycle. The rat/mouse follicular phase (2–3 days) is called diestrus. The periovulatory phase of the rat and mouse is called proestrus and occurs during the day of E2 and progesterone surges. The estrus phase of the rat/mouse cycle occurs on the day following the E2 and progesterone surges; this is when the female rodent ovulates and is sexually behaviorally receptive (Becker et al., 2005).

### Cocaine

It is well established that compared to males female rats demonstrate more robust operant behavior during acquisition of cocaine self-administration, escalation of drug intake, and reinstatement of extinguished drug-taking behavior (Lynch and Carroll, 1999, 2000; Roth and Carroll, 2004). Moreover, female rats acquire cocaine self-administration more quickly and at lower doses than males (Lynch and Carroll, 1999; Davis et al., 2008).

Estrous cycle phase has been shown to influence an animal’s motivation to self-administer cocaine (Roberts et al., 1989); cocaine-self administration is highest during proestrus and estrus and lowest during diestrus (Feltenstein et al., 2011). In other words, female rats consume greater amounts of cocaine when circulating levels of E2 are high and cocaine consumption is reduced at times when E2 levels are lower. The notion that different levels of circulating ovarian hormones are important for differences in the reinforcing properties of drugs is further supported by self-administration paradigms using OVX-hormone-treated rodents. Such experiments have consistently demonstrated a role for E2 in enhancing the responsivity to cocaine. More specifically, ovariectomy alone decreases the rate of acquisition of cocaine self-administration and reinstatement of previously extinguished cocaine-seeking behavior. E2 administration to OVX animals restores acquisition of cocaine self-administration to levels comparable with those of intact female rodents (Lynch et al., 2001; Hu et al., 2004; Larson et al., 2005; Frye, 2007). Furthermore, this effect was specific for female rodents since there was no effect of E2 replacement on cocaine self-administration behaviors of male rodents (Jackson et al., 2006).

Continued drug use, drug-seeking and relapse to former patterns of drug use during abstinence are heavily dependent on learning associations between the drug and environmental cues and/or contexts as well as the individual’s physical and emotional reactivity to these stimuli (O’Brien et al., 1990; Enmark et al., 1997). Susceptibility of females to cocaine-associated cues and contexts is an important underlying factor in the sex-differences seen in cocaine addiction (Robbins et al., 1999). The conditioned place preference paradigm (CPP) is used to determine the conditioned rewarding effects of drugs in rodents because the contextual (environmental) cues used within the paradigm acquire secondary appetitive properties when paired with a rewarding stimulus (i.e., drug of abuse) (Bardo and Bevins, 2000; Tzschentke, 2007). Additionally, the cues/context itself acquires rewarding properties that are directly associated with the subjective rewarding effects of the drug. Sex differences in the rewarding properties of cocaine have been well illustrated using this paradigm by our group and others. Specifically, female rats demonstrate acquisition of conditioned place preference to lower doses of cocaine compared to males (Russo et al., 2003b; Zakharova et al., 2009). This indicates that at lower doses females experience an increase in the magnitude of the rewarding effects of cocaine when compared to males (Russo et al., 2003b; Zakharova et al., 2009). However, at higher doses, cocaine place preference is similar between males and females. This suggests that female sensitivity to cocaine’s effects is dose dependent and maybe acutely enhanced at lower doses. Circulating levels of E2 influence the magnitude of CPP, however, these effects vary according to the dose and length and time course of hormone treatment. We and others have reported that OVX female rats demonstrate lower CPP scores compared to intact females (Russo et al., 2003a; Kokane et al., 2019). In a series of experiments in which chronic continual administration of E2 was given throughout the duration of the CPP paradigm, E2 treatment alone did not influence CPP when compared to untreated animals (Russo et al., 2003a). Recent studies from our laboratory, demonstrate that low dose E2 administration to OVX female rats during the conditioning phase of CPP enhances expression of cocaine place preference (Kokane et al., 2019). Conversely, a single injection of E2 prior to the test phase of cocaine CPP, blunted the expression of cocaine place preference in OVX rats (Bobzean et al., 2014a). This finding indicates that elevations in E2 after drug-associations have taken place, my actually serve to inhibit the conditioned response or decrease the salience of the stimulus. Taken together these data elaborate the influence of E2 on the rewarding and reinforcing properties of cocaine. It is also evident from the above studies that the timing of the elevations in E2 levels is important toward these effects.

### Morphine

Overall, female rodents acquire both oral and IV self-administration of opioids faster than males, at lower doses, and under a wider variety of environmental housing factors (Alexander et al., 1978; Lynch and Carroll, 1999; Cicero et al., 2003). Moreover, females consume greater amounts of opioids and will work harder for a dose of an opioid drug than males (Cicero et al., 2003). In tests of CPP, female mice demonstrate preferences for environments associated with lower doses of morphine compared to males (Karami and Zarrindast, 2008). Taken together, these experiments have identified sex differences in opiate reward and reinforcement. In addition, there are limited data suggesting that E2 enhances the salience of cues paired with opioid reward (CPP) (Roth et al., 2002; Mirbaha et al., 2009). Therefore, these findings suggest that E2 augments the rewarding/reinforcing properties of opioids and that fluctuating levels of E2 in females may increase (or decrease) their vulnerability to opioid taking behaviors.

## Sex Differences in the Neurobiology of Addiction

Sex differences in behavioral effects of cocaine are largely the result of underlying neurobiological changes brought about by the interactions between cocaine and ovarian hormones within reward-related circuitry. Although specific neurobiological mechanisms remain to be fully elucidated, there is much evidence implicating the combined effect of cocaine and E2 on VTA DAergic neurotransmission and on striatal GABAergic, DAergic, and glutamatergic neurotransmission. Additionally, it has been demonstrated that drug use, whether it be cocaine or opiates, produces persistent enhanced activation of the mesolimbic reward circuit resulting in long-term structural and functional changes. These neuroadaptive changes include enhancement in neurotransmitter release, increased synaptic plasticity and dendritic arborization within areas of the mesolimbic reward pathway. The increased neurotransmission (GABAergic, dopaminergic, and glutamatergic) activates downstream molecular mechanisms within these areas. Estradiol has been shown to modulate the activity of these molecular mechanisms. In this section, we discuss the influence of E2 on the molecular mechanisms which underlie the persistent effects of cocaine and morphine on the structural and functional changes in the mesolimbic reward system.

### Cocaine

Cocaine exerts its psychomotor stimulant effects by increasing extracellular DA levels by binding to the dopamine transporter (DAT) in the striatum; a membrane protein located on nerve terminals responsible for the reuptake of DA from the synaptic cleft (Mortensen and Amara, 2003; Zhu and Reith, 2008). Through this inhibition of DA reuptake, cocaine increases synaptic DA levels thereby potentiating activation at postsynaptic DA receptors. Cocaine produces a buildup of DA wherever the brain has DA transporters. However, the psychoactive and addictive effects of cocaine are generated by the drug’s ability to produce a buildup of DA in mesolimbic reward structures (Koob et al., 1998; Hyman and Malenka, 2001; Nestler, 2001; Kalivas and McFarland, 2003).

Activation of G-protein-coupled D1 receptors initiates a cellular signaling cascade that enhances phosphorylation of the transcription factor cAMP response element binding protein (CREB) and expression of immediate-early genes (Nestler, 2002; Walters et al., 2003). Generally speaking, the positive rewarding effects of cocaine are mediated via D1-MSNs (Hikida et al., 2010; Lobo et al., 2010; Lobo and Nestler, 2011; Bock et al., 2013; Chandra et al., 2013; Lenz and Lobo, 2013) and many cocaine-induced molecular adaptations occur within these D1 MSNs (Lobo and Nestler, 2011; Grueter et al., 2013; Lobo et al., 2013). The increases in cAMP in response to D1 receptor stimulation initiate activation of downstream protein kinases, including protein kinase A (PKA) and extracellular signal-regulated kinase (ERK). These kinases, along with many other downstream effects, can phosphorylate the transcription factor CREB (Kano et al., 1995). CREB activation in the NAc is essential as a regulator of cocaine reward and for cocaine-dependent alterations in gene expression (McClung and Nestler, 2003; Lemberger et al., 2008; Renthal et al., 2009). Post synaptic D2 receptors either inhibit or have no effect on adenylyl cyclase activity; presynaptic D2 receptors function as auto-receptors (Clark and White, 1987; Meador-Woodruff et al., 1991; Surmeier et al., 2007). Generally speaking, D1 receptors play a role in the primary rewarding properties of cocaine, while D2 receptors play a role in drug-seeking mechanisms (Self et al., 1996; Lobo and Nestler, 2011). The repeated administration of cocaine causes adaptations in such DA receptor stimulated signaling pathways which manifest into the behaviors characteristic of cocaine addiction (Anderson and Pierce, 2005; McGinty et al., 2008; Thomas et al., 2008).

#### Overview of Signaling Mechanisms of Cocaine Addiction

Molecular mechanisms associated with cocaine reward, reinstatement, craving and dependence involve extracellular signal regulated kinase (ERK), calcium/calmodulin-dependent kinase II (CaMKII), protein kinase C (PKC), cAMP-dependent protein kinase A (PKA), cGMP-dependent protein kinase G (PKG), phosphatidylinositol 3-kinase (PI3K) and its downstream target mammalian target of Rapamycin (mTOR), cyclin-dependent kinase 5 (Cdk5), transcription factors (cAMP response element binding protein – CREB, nuclear factor-kappa B – NF-κB, delta Fos B – ΔFosB) and brain derived neurotrophic factor (BDNF).

CREB, NF-κB, and ΔFosB are transcription factors regulating the expression of several genes involved in synaptic plasticity, neuroadaptations dendritic arborization and have been implicated in the development of cocaine addiction-related behaviors. CREB gets phosphorylated by PKA, CaMKII and ERK signaling pathway. Increased phosphorylation of CREB in the NAc, blunts the rewarding effects of cocaine thereby driving cocaine self-administration (Carlezon et al., 1998; Barrot et al., 2002; Larson et al., 2011). Through empirical evidence, it has been proposed that increased activity of CREB leads to increased excitability of NAc MSNs via increased expression and synaptic transmission of NMDARs. This may in turn induce a negative feedback loop that blunts rewarding effects of cocaine and eventually drives escalation (Dong et al., 2006). Phosphorylation of CREB also drives formation of new dendritic spines by increasing the expression of NMDARs but not AMPARs leading to the formation of “silent synapses” (Murphy and Segal, 1997; Segal and Murphy, 1998). Studies have indicated formation of “silent synapses” to be critical to the enhancement of cocaine seeking (Huang et al., 2015). NF-κB is upregulated in NAc post chronic cocaine administration (Ang et al., 2001). It is involved in expression of cocaine reward and the induction of dendritic spines in the MSNs of NAc (Russo et al., 2009). Genes transcribed by NF-κB are essentially required in the regulation of synaptic plasticity of neurons within the mesolimbic reward regions (Engelmann and Haenold, 2016). ΔFosB is induced in the D1-type MSNs by chronic exposure to cocaine and has been proposed to be the molecular switch for addiction (Nestler, 2008; Perrotti et al., 2008). Its expression is regulated by cocaine and CREB within NAc MSNs and increases cocaine reward and self-administration (Nye et al., 1995; Vialou et al., 2012; Robison et al., 2013). It is also necessary and sufficient for cocaine-induced dendritic spine formation through increase in the expression of “silent synapses” in the D1-type MSNs of NAc. Conversely, it decreases their expression in D2-type MSNs of the NAc thereby enhancing rewarding effects of cocaine (Grueter et al., 2013). It has been shown to be critical in mediating the effects of cocaine on NAc’s ability to integrate glutamatergic inputs from the hippocampus, mPFC and amygdala (Eagle et al., 2019).

#### Sex Differences in Signaling Mechanisms of Cocaine Addiction

Sex differences in baseline and cocaine-induced activation of cAMP and PKA pathways in the NAc have been documented. Overall, female rats exhibit higher levels of total PKA protein and phosphorylated DARPP-32 in the NAc compared to males, regardless of drug-treatment condition (drug naïve, saline-treatment, or cocaine-treatment) (Lynch et al., 2007; Nazarian et al., 2009). Moreover, males and females have different basal and cocaine-induced levels of pERK, ΔFosB, and pCREB in the NAc (Nygard et al., 2013). It is possible that this may be due to the differences in DA receptor expression and binding (Yoest et al., 2018). Studies have also shown E2 regulation of cAMP and PKA pathways. For example, the estrous cycle seems to affect the activity of a variety of intracellular signaling cascades in the NAc of female rats regardless of cocaine-treatment (Weiner et al., 2009). Ovariectomized females have been used in attempts to identify a role of E2 on these intracellular signaling cascades. The results of one study show that OVX E2 treated females demonstrate E2-induced initiation of PKA cascades and CREB protein phosphorylation via activation of G-protein dependent cell signaling cascades (Hammes and Levin, 2007). The downstream effects of these results likely contribute to the structural sexual dimorphisms seen in dendritic morphology and spine density (Forlano and Woolley, 2010; Wissman et al., 2011, 2012).

#### Estrogen Receptors Influence Cocaine-Induced Intracellular Signaling

It is well established that estrogens produce their effects by genomic and non-genomic actions. The so-called “genomic or classical estrogen receptors” are ligand-activated transcription factors which reside in the cytosol and translocate to the nucleus upon ligand binding and dimerization (Nilsson et al., 2001). As with other steroid hormone receptors, ERs can either modulate gene expression directly, by binding to consensus target DNA sequence, or indirectly, by interacting with other transcription factors to activate or repress gene activation. Estrogen also has acute, rapid (non-genomic) effects, which are initiated via binding at plasma associated membrane estrogen receptors (mER) (Boulware et al., 2005; Mermelstein and Micevych, 2008; Micevych and Mermelstein, 2008). Signaling at mERs activates G-protein dependent cell signaling cascades (Hammes and Levin, 2007), including PKA and MAPK (Dhandapani and Brann, 2002; Bjornstrom and Sjoberg, 2005; Ronnekleiv et al., 2007; Vasudevan and Pfaff, 2007). These signaling cascades are similar to those initiated by DA at D1 receptors. In fact, evidence for the role for mERs in mediating the rapid effects of E2 stems from its effects on CREB phosyphorylation (pCREB). ERα and ERβ antagonists mimic the effects of E2 while the mER antagonist ICI 182,780 blocks the rapid effects of E2 on pCREB. In this way, E2 activates both mER and D1 receptor G-protein-dependent cell signaling cascades including activation of the MAPK pathway, and phosphorylation of CREB (Hammes and Levin, 2007).

Adult female rats predominantly express membrane-bound ERs (GPER1, membrane associated ERα and ERβ) in MSNs of the DS and NAc, but express few or no nuclear ERs (Mermelstein et al., 1996; Kuppers et al., 2008; Schultz et al., 2009; Grove-Strawser et al., 2010; Almey et al., 2012, 2015). Membrane ERs are expressed on axon terminals, somas, and dendritic spines (Almey et al., 2012, 2015, 2016). Functionally, the activation of MSN membrane ERs have been shown to increase sensitivity to drugs of abuse in females (Tonn Eisinger et al., 2018) and change dendritic spine morphology and density in the NAc (Peterson et al., 2015). Previous work has established that application of E2 rapidly increases DA (Becker, 1990; Pasqualini et al., 1996) and decreases GABA production (Hu et al., 2006) in the NAc and DS which suggests that E2 may indirectly influence DA signaling by first releasing inhibition of GABAergic signaling, and perhaps also directly upon DA-producing regions (such as the VTA). In striatal MSNs, E2, acting through membrane-associated ER alpha and beta receptors coupled to mGluRs modulates phosphorylation of the transcription factor CREB (Mermelstein et al., 1996; Grove-Strawser et al., 2010).

Similar to the DS, E2 also rapidly modulates glutamate signaling in NAc core and these effects are sex-specific and bidirectional (Krentzel et al., 2019). The mechanism whereby E2 enhances drug-induced plasticity is via interactions with mGluRs. Specifically, E2 activates mGluR5 signaling in the NAc core, which in turn, leads to alterations in dendritic structure (Grove-Strawser et al., 2010; Martinez et al., 2014; Peterson et al., 2015). These alterations induce neuroadaptations which are long-lasting and cause long-term enhanced activation of the mesolimbic reward pathway. As a result, activation of the reward pathway and DA release in response to “naturally” rewarding stimuli is insufficient. Hence, normal rewarding stimuli become less rewarding and full activation of the reward pathway requires drug consumption.

Estradiol and DA systems interact to modulate striatal function and resultant behavior (Di Paolo et al., 1985; Becker and Beer, 1986; Becker, 1990; Bitar et al., 1991). For example, E2 increases striatal DA release and turnover (Becker and Ramirez, 1981b; Becker et al., 1984; Di Paolo et al., 1985; Becker and Beer, 1986) and density of striatal DA uptake sites (Morissette et al., 1990). Post-synaptically, E2 increases striatal D1 receptors, while decreasing high-affinity D2, and increasing low-affinity D2 binding (Di Paolo et al., 1985; Levesque and Di Paolo, 1988, 1989; Morissette et al., 1990; Shieh and Yang, 2008). Presynaptically, E2 potentiates amphetamine-induced DA release and turnover in the NAc (Becker et al., 1984; Di Paolo et al., 1985; Thompson and Moss, 1994). Additionally, E2 promotes the sensitivity of VTA-DA neurons to cocaine (Zhang et al., 2008) which, in turn, enhances cocaine-stimulated release of DA in the striatum (Peris et al., 1991; Febo et al., 2003). Therefore, the potentiating effects of E2 on striatal DA activity are, in part, a cause of the sex and hormone-related differences in subjective and physiological responses to cocaine (Becker and Ramirez, 1981b; Becker and Rudick, 1999; Walker et al., 2001; Quinones-Jenab, 2006). The effect of E2 in the female striatum is also mediated by E2 receptors on GABAergic MSNs that enhance DA release via disinhibition of local DAeric terminals (Mermelstein et al., 1996; Mermelstein, 2009; Schultz et al., 2009; Grove-Strawser et al., 2010). Thus effects of E2 on cocaine self- administration and CPP may be due to the ability of E2 to act on mesolimbic DA system to regulate reward and motivation, through the ability of E2 to increase cocaine-stimulated DA release in NAc (Tobiansky et al., 2016) and alter signaling pathways and gene expression in striatum (Le Saux et al., 2006; Grove-Strawser et al., 2010; Peterson et al., 2015, 2016).

Interactions between E2 and cocaine have been demonstrated in that E2 enhances the sensitivity of the DAergic neurons of the VTA to cocaine and increases cocaine-induced DA release in the striatum (Peris et al., 1991; Zhang et al., 2008). A recent study by Calipari et al. (2017) demonstrated that female mice in estrus showed increased cocaine-induced DA release compared to female mice in diestrus or male mice. Indeed, these neurobiological effects resulted from greater firing rate of VTA-DAergic projections and DA release in the NAc of female mice in estrus (Zhang et al., 2008; Calipari et al., 2017). Moreover, these estrous females also displayed increased phosphorylation of dopamine transporter (DAT) protein. Taken together, it can be hypothesized that higher levels of circulating E2 increase DA levels in the NAc which is due to increased inhibition of DAT activity under the influence of cocaine (Calipari et al., 2017).

E2 stimulation of mERs rapidly stimulates MAPK-dependent pCREB, and peripheral administration of E2 initiates MAP kinase and PKA signaling pathways (Mhyre and Dorsa, 2006; Dewing et al., 2007, 2008; Kelly and Ronnekleiv, 2008) and decreases L-type calcium channel-mediated CREB activity (Gu et al., 1996; Zhou et al., 1996; Boulware and Mermelstein, 2005; Boulware et al., 2005, 2007). Inhibition of either MEK or PKC significantly inhibits E2-mediated DA efflux, while inhibiting PI3 kinase or PKA does not affect E2-mediated DA efflux (Alyea and Watson, 2009). E2 induction of TH involves membrane-initiated E2 signaling, rapid activation of dual PKA/MEK signaling pathways, leading to pCREB activity (Maharjan et al., 2010), while the mER antagonist ICI 182,780 blocks the rapid effects of E2 on pCREB. Thus, mER mediated activation of these intracellular signaling cascades influences the activity of a variety of transcription factors which likely contribute to gene transcription independently of nuclear ER (Mhyre and Dorsa, 2006).

Although extensive research in male animals has attributed several different intracellular signaling cascades to different processes associated with cocaine-addiction, studies of these same intracellular signaling mechanisms in females is almost non-existent. However, based on the above discussion, estradiol and cocaine separately or together, affect all of the aforementioned signaling molecules/transcription factors. Further research demonstrating the influence of estrogen/estradiol on the function/activity of these signaling molecules, transcription factors and BDNF is essentially required.

### Morphine

Morphine and other opioids exert their rewarding effects through stimulation of mu opiate receptors (MORs) localized at the GABAergic terminals in the VTA (Johnson and North, 1992a; Bonci and Williams, 1997). Opioid activation of MORs disinhibits VTA-GABA neurons, which in turn, increases the release of DA to the NAc (Pontieri et al., 1995; Lecca et al., 2007). The increased release of DA in the NAc induces feelings of euphoria and promotes the development of drug dependence (Wise and Rompre, 1989; Johnson and North, 1992b; Bodnar, 2014).

#### Overview of Morphine Addiction Mechanisms

The MOR is a conventional G-protein coupled receptor in that it is a cell surface protein with seven transmembrane domains consisting of α, β, and γ subunits, and an effector protein. Upon activation by an agonist, the Gα and Gβγ subunits dissociate from one another and subsequently regulate a variety of intracellular effector pathways. In case of the VTA-GABAergic interneurons, morphine activation of MORs, suppresses the release of GABA within the VTA thereby promoting DA release from the DAergic projections arising from the VTA. Acute morphine treatment followed by prolonged abstinence produces burst firing of VTA-DA neurons which is believed to play a role in encoding reward value (Schultz, 2002; Jalabert et al., 2011; Fields and Margolis, 2015). Chronic exposure to morphine results in compensatory changes in the cell that oppose the initial alterations observed after acute exposure. Chronic morphine treatment has been shown to reduce the size of VTA-DA neurons (Russo et al., 2007) and increase basal firing rate (Koo et al., 2012) in male rats thus upregulating levels of DA in VTA-NAc circuits (Beitner-Johnson et al., 1991). Therefore, the rewarding effects of opioids are linked to the drug’s ability to activate the mesolimbic DA pathway (Di Chiara and Imperato, 1988; Pierce and Kumaresan, 2006).

**FIGURE 3:**
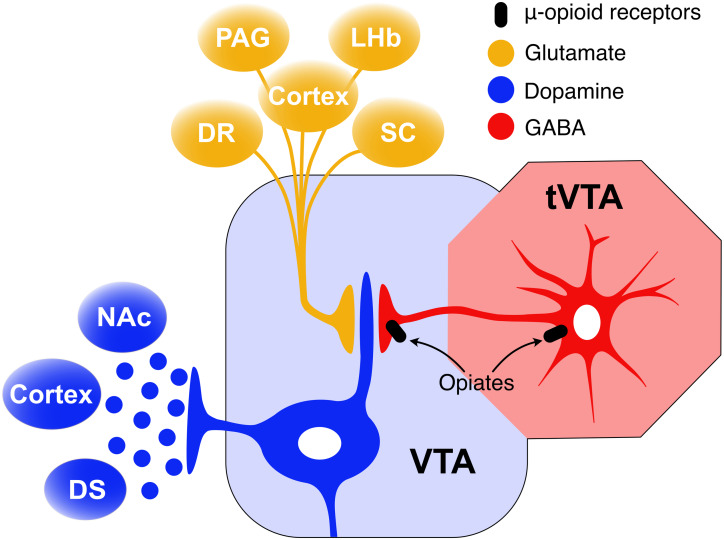
GABA inhibition from the tVTA/RMTg mediates VTA-DA excitation. The main input of the tVTA is a glutamatergic projection from the lateral habenula (LHb) while its main output is a GABAergic projection to the VTA. tVTA-GABAergic neurons are densely packed with mu opioid receptors the activation of which decreases their firing rate. As a result, DA neurons in the VTA are disinhibited and freely transmit DA to projection sites in the NAc, DS, and cortex.

The VTA receives inputs from the tail of the VTA (tVTA)(Perrotti et al., 2005)/rostromedial tegmental nucleus (RMTg) (Jhou et al., 2009a) and the NAc (Figure 3). This input is believed to modulate the activity of VTA-DA neurons (Koo et al., 2012; Tan et al., 2012; van Zessen et al., 2012; Taylor et al., 2016). Studies using male rodents show that opioid drugs induce rewarding effects through inhibition of GABAergic interneurons, leading to excitation of VTA-DA neurons (Gysling and Wang, 1983; Johnson and North, 1992b). This VTA-DA excitation is mediated via GABAergic projections from the tVTA/RMTg. The tVTA/RMTg, caudal to the VTA, is a GABAergic area innervating VTA-DA cells (Bourdy et al., 2014). MORs are densely packed on these tVTA-GABAergic neurons (Jhou et al., 2009b, 2012; Jalabert et al., 2011; Kaufling and Aston-Jones, 2015), and MOR binding decreases their firing rate (Jalabert et al., 2011; Kaufling and Aston-Jones, 2015). Consequently, DA neurons in VTA are disinhibited and freely transmit DA to projection sites in the NAc and other limbic regions which leads to overstimulation of the circuitry mediating addiction-related behaviors. Acute morphine treatment activates VTA-DA neurons via inhibition of GABAergic projections from the tVTA/RMTg (Lecca et al., 2012; de Guglielmo et al., 2015; Kaufling and Aston-Jones, 2015). Naloxone precipitated withdrawal from chronic continual morphine treatment has been shown to increase release of GABA in the tVTA of male rats (Kaufling and Aston-Jones, 2015). Although there is no single brain region responsible, a growing body of evidence supports morphine withdrawal-induced increases in tVTA GABA release in triggering the intracellular events that manifest aspects opiate withdrawal syndrome (Madhavan et al., 2010; Kaufling and Aston-Jones, 2015).

The molecular mechanisms underlying opioid reward and dependence are under the direct control of G-protein activation including adenylyl cyclases, ion channels, and components of the mitogen activated protein kinase (MAPK) cascade. For example, agonist stimulation of opioid receptors leads to an inhibition of adenylate cyclase activity and a reduction of cyclic adenosine monophosphate (cAMP) levels in the cell (Sharma et al., 1977; Chakrabarti et al., 2016) as well as a suppression of the activity of protein kinase A (Fleming et al., 1992; Zhang and Pan, 2010). In addition, the Gα subunit directly interacts with G-protein inward rectifying potassium channels leading to increased hyperpolarization of the cell, and reduced cell excitability (North et al., 1987; Law et al., 2000). The dissociated Gβγ subunit is responsible for the direct blockade of calcium channels therefore reducing intracellular calcium concentrations (Moises et al., 1994), which leads to suppression neurotransmitter release.

Following removal of the opiate, the functional activity of the cAMP pathway increases continuously. These changes in the functional state of the cAMP pathway are regulated through stimulation of adenylyl cyclases and protein kinase A as a consequence of chronic administration of opiates. The phosphorylated cAMP response element (CRE)-binding protein (pCREB), and related proteins, are indicators of protein kinase A activity. Studies in male rodents indicate that CREB is essential for morphine-induced changes in gene expression precipitating reward- and withdrawal-associated behavior: opioid withdrawal upregulates cAMP by increasing adenylyl cyclase activity, leading to increased pCREB levels in reward-related brain regions.

#### Sex Differences in the Neurobiological Response to Opiate Reward

Sex-based differences in the cellular and neurobiological mechanisms underlying sex differences in the rewarding properties of opioids are poorly understood (Dahan et al., 2008; Lee and Ho, 2013; Huhn et al., 2018). Converging evidence from the reproductive and pain literatures suggests interactions among opioids, gonadal hormones, opioid receptors, and estrogen receptors may underlie sex differences in addictive responsiveness to opioid drugs (Lee and Ho, 2013).

There is evidence demonstrating sex differences in the development of tolerance and dependence to morphine (Craft et al., 1999, 2004; Dahan et al., 2008). Sex differences in the potency of morphine seem to account for differences in tolerance when animals are exposed to the same dose of morphine (Barrett et al., 2001). However, most of what is currently known about opioid dependence and withdrawal comes from studies conducted exclusively in males. Results of the few sex differences studies that have been conducted show that male rodents express a greater magnitude of withdrawal symptoms than females during spontaneous withdrawal from chronic morphine administration (Cicero et al., 2002), but not naloxone-precipitated withdrawal (Ali et al., 1995; Cicero et al., 2002). However, caution should be taken when evaluating the methods and results of these experiments and others; at this point in time, most withdrawal scales have been developed using only male animals. As such, a detailed withdrawal syndrome for the female phenotype is currently unknown.

Recently, work from our lab has begun to establish sex differences in the behavioral response and molecular signaling within the tVTA resulting from morphine withdrawal (Bobzean et al., 2019). We observed sex differences in the expression and duration of spontaneous somatic morphine withdrawal. Morphine dependent male rats displayed a more severe opiate withdrawal syndrome sooner after cessation of morphine, while females displayed a more protracted withdrawal syndrome which lasting at least 72h after termination of morphine treatment (Bobzean et al., 2019). In addition, we demonstrated a correlation between activation of CREB in the tVTA and severity of morphine withdrawal symptoms in females (Bobzean et al., 2019). Overall, these findings indicate that ovarian hormone status may influence the severity and/or persistence of symptoms and CREB expression.

Sex differences and hormonal regulation of opioid receptor binding and density as it relates to reproductive behaviors are well known. Several studies have identified sex differences and hormonal regulation of opioid receptor densities (Limonta et al., 1991; Maggi et al., 1991, 1993; Dondi et al., 1992; Piva et al., 1995), distribution, and signaling efficiency (Lee and Ho, 2013; Huhn et al., 2018). There is evidence demonstrating sex differences in the development of analgesic tolerance and dependence to morphine (Craft et al., 1999, 2004; Dahan et al., 2008). Sex differences in the potency of morphine seem to account for differences in tolerance to the antinociceptive effects of the drug when animals are exposed to the same dose of morphine (Barrett et al., 2001). Although males develop analgesic tolerance to chronic morphine administration more quickly than females, one study observed higher NAc glutamate levels in morphine-tolerant female rats (Mousavi et al., 2007). Increased glutamate in NAc induces tolerance by downregulating glutamate transporters (Yang et al., 2008).

In summary, studies of sex differences in opioid withdrawal are severely lacking, and the few that do exist report inconsistent results: male rodents express greater somatic symptoms of opioid withdrawal than females (Cicero et al., 2002; Towers et al., 2019); females are more sensitive than males (Ali et al., 1995; Papaleo and Contarino, 2006); no sex differences, or females more severely affected after acute morphine administration or naloxone-precipitated withdrawal (el-Kadi and Sharif, 1994; Ali et al., 1995; Craft et al., 1999; Cicero et al., 2002; Papaleo and Contarino, 2006; Bodnar and Kest, 2010). Aside from methodological differences (i.e., drug dose, exposure frequency/duration, withdrawal sign measured, etc.), these inconsistencies may be due, in part, to lack of assessment of estrous cycle phase in females (Bobzean et al., 2014b; Becker and Chartoff, 2019). Currently, most of what is currently known about opioid withdrawal has been derived from studies exclusively in males and then extrapolated to females as most withdrawal scales have been developed using male subjects (Arfken et al., 2001; Puigdollers et al., 2004). The dearth of studies characterizing opioid withdrawal in females is surprising, given reports that sex and ovarian hormone activity modulate pain sensitivity (Berkley, 1997; Gureje et al., 1998; Stoffel et al., 2003; Fillingim et al., 2009; Green et al., 2009; Mogil, 2012), pain inhibition (Negus et al., 2002; Craft, 2003; Stoffel et al., 2003), nociception processing/opioid analgesia (Mogil, 2012; Amandusson and Blomqvist, 2013), and sensitivity to stress (Hodes, 2018), all variables known to promote opioid use.

## Summary

The evidence identifying important differences in the patterns of drug use, abuse, and addiction between men and women implicates sex as a risk factor for developing substance use disorder and as a powerful component affecting the course of treatment. Sex differences and sensitivity to gonadal hormones within the structure and functions of the mesolimbic reward system are linked to sex differences in the neurobehavioral response to drugs of abuse. It has also been demonstrated that the underlying cause of these sex differences on DA signaling is the cyclic fluctuation in E2 levels and its downstream neurobiological effects.

The overarching goal of much of the work reviewed in this paper is to determine how hormonal cycles affect the development of addictive phenotypes. Beginning in the 1990s studies identified estrous cycle effects on DA release and uptake in the NAc and DS (Thompson and Moss, 1994; Xiao and Becker, 1994; Thompson and Moss, 1997; Cecconi et al., 2004). Since then, data have showed that striatal DA release is increased during estrous cycle phases associated with high levels of E2 (Yoest et al., 2018). Moreover, DA clearance is shown to be substantially lower during these same phases of the cycle and higher during phases with low levels of circulating E2 (Yoest et al., 2018). In addition, basal firing rates of DA neurons of the VTA vary during the different phases of the rodent estrous cycle: DA firing rates are highest in estrus, lowest in proestrus, and intermediate in diestrus (Zhang et al., 2008). More recent work, has confirmed and extended these findings by demonstrating high levels of E2 enhance DAergic responses to cocaine and cocaine-associated rewarding cues (Calipari et al., 2017). This, in turn, leads to increased VTA-NAc responses to the drug-associated cues alone even at later stages of the cycle (Calipari et al., 2017). Moreover, cues paired with cocaine during estrus results in greater activation of striatal brain regions (Johnson et al., 2019). Taken all together, these data demonstrate a potential mechanism by which drug-induced potentiation of the activity of DA projections from the VTA to the NAc during estrus could motivate self-administration behavior and increased responsitivity to drug-associated cues during other phases of the cycle.

Over 30 years of research has established that cyclic fluctuations of E2 modulate the mesolimbic reward. Parallel research demonstrates that mesolimbic reward pathways become dysregulated under states of chronic drug use. As these two areas of research converge, it becomes readily apparent that fluctuations in levels of E2 over the hormonal cycle of the female likely modulate the drug-induced dysregulation of the mesolimbic DA reward pathway over the course of individual’s drug taking career. Therefore, when considering sex differences in addiction, it is critical to consider female hormonal cycles.

## Author Contributions

SK and LP participated in drafting various sections of the manuscript and revising it critically for important intellectual content. LP gave final approval of the version to be submitted.

## Conflict of Interest

The authors declare that the research was conducted in the absence of any commercial or financial relationships that could be construed as a potential conflict of interest.
